# Changes of bladder urodynamics after combined pelvic floor muscle training and intravaginal stimulation in women with stress urinary incontinence: a retrospective cohort study

**DOI:** 10.3389/fbioe.2026.1869813

**Published:** 2026-08-31

**Authors:** Hui-Hsuan Lau, Cheng-Yuan Lai, Ming-Chun Hsieh, Hsien-Yu Peng, Dylan Chou, Tsung-Hsien Su, Jie-Jen Lee, Tzer-Bin Lin

**Affiliations:** 1 Division of Urogynecology, Department of Obstetrics and Gynecology, Mackay Memorial Hospital, Taipei, Taiwan; 2 Department of Nursing, Mackay Junior College of Medicine, Nursing, and Management, Taipei, Taiwan; 3 Department of Medicine, Mackay Medical College, Taipei, Taiwan; 4 Institute of Biomedical Sciences, Mackay Medical College, New Taipei, Taiwan; 5 Department of Surgery, Mackay Memorial Hospital, Taipei, Taiwan; 6 Institute of Translational Medicine and New Drug Development, College of Medicine, China Medical University, Taichung, Taiwan; 7 Department of Physiology, School of Medicine, College of Medicine, Taipei Medical University, Taipei, Taiwan; 8 Graduate Institute of Biomedical Electronics and Bioinformatics, National Taiwan University, Taipei, Taiwan

**Keywords:** biofeedback, electric stimulation, pelvic floor strengthening, urodynamics, voiding, workload

## Abstract

**Introduction:**

Sling implantation increases bladder outlet resistance and consequently elevates voiding workload in patients with stress urinary incontinence (SUI). This study investigated whether a non-invasive approach—pelvic floor muscle strengthening combined with intravaginal stimulation (PFMT + IVS)—modulates voiding work through alterations in outlet resistance.

**Methods:**

Cystometric data from 20 female patients with SUI who completed a home-based PFMT + IVS program were retrospectively analyzed. The intervention consisted of biofeedback-assisted pelvic floor muscle training and intravaginal electrical stimulation (20 min each, twice weekly for 3 months). Symptom severity and quality of life were assessed using the Urogenital Distress Inventory–6 (UDI-6) and Incontinence Impact Questionnaire–7 (IIQ-7). Urodynamic parameters and pressure–volume analysis (PVA) indices were evaluated before and after treatment.

**Results:**

Following PFMT + IVS, patients showed significant reductions in UDI-6 (p < 0.001; N = 20) and IIQ-7 scores (p < 0.001; N = 20). Voided volume (Vvoid; p = 0.042), voiding time (Tvoid; p = 0.028), and the pressure–volume trajectory area (Apv; p = 0.044), a surrogate of voiding work, were significantly increased. In contrast, no significant changes were observed in voiding resistance (Rvoid; p = 0.089), voiding pressure (Pvoid; p = 0.306), or flow rate (Fvoid; p = 0.271). The increase in Apv correlated positively with the increase in Vvoid (r = 0.450, p = 0.046), and the increase in Vvoid correlated with prolonged Tvoid (r = 0.665, p = 0.001).

**Conclusion:**

PFMT + IVS appears to maintain adequate outlet resistance during storage while permitting effective relaxation during voiding, thereby preserving voiding pressure and flow. Although total work per voiding cycle increased, work normalized to voided volume remained unchanged, suggesting preserved voiding efficiency.

## 1 Introduction

Stress urinary incontinence (SUI), resulting from weakness of the urethral sphincter or its supporting structures, is defined by involuntary urine leakage during activities such as coughing, sneezing, or physical exertion (Abrams et al., 2003). In older women, particularly those over 70 years of age, SUI significantly impairs daily functioning, hygiene, and social participation (Abrams et al., 2002). Pelvic floor muscle training (PFMT) is recommended as the first-line conservative treatment (National Institute for Health and Care Excellence, 2019), whereas minimal invasive options including urethral bulking and midurethral slings is reserved for patients who do not respond to conservative therapy (Gallo et al., 2024). Both non-invasive (Todhunter-Brown et al., 2022) and minimal invasive (Särkilahti et al., 2025) approaches have been shown to improve continence and quality of life (QoL). However, sling procedures increase urethral resistance, which elevates voiding pressure and consequently increases voiding workload (Arlandis, 2025). Given that chronic bladder overload may contribute to detrusor decompensation (Chow et al., 2021), it is clinically important to determine whether non-invasive therapies exert similar effects on voiding work.

PFMT is widely used as the cornerstone of conservative SUI management (Peyronnet et al., 2025; Elser, 2012). Biofeedback-assisted PFMT enhances patient awareness and performance of pelvic floor muscle contractions (Hagen et al., 2020; Herderschee et al., 2011; Ladi-Seyedian et al., 2019) and has been shown to improve continence outcomes (Todhunter-Brown et al., 2022). In parallel, intravaginal electrical stimulation (IVS), an established modality for muscle strengthening, directly activates pelvic floor muscles via a vaginal electrode and serves as an additional non-invasive treatment option (Todhunter-Brown et al., 2022; Porcari et al., 2002). As both interventions are amenable to home-based application, their combined use (PFMT + IVS) has been explored to maximize therapeutic efficacy, with evidence demonstrating improvements in bladder storage function (Lau et al., 2024a). However, the effects of PFMT + IVS on voiding function, particularly voiding workload, remain unclear.

To address this gap, cystometry-derived pressure–volume analysis (PVA) (Lau et al., 2023; Lau et al., 2024b; Lau et al., 2024c; Lau et al., 2026; Peng et al., 2020; Peng et al., 2021) was employed. In PVA, the trajectory-enclosed area (Apv), a surrogate measure of voiding energy expenditure, was quantified to evaluate changes in voiding workload before and after PFMT + IVS.

## 2 Methods

### 2.1 Patients

This study was conducted in accordance with the Declaration of Helsinki and was reviewed, approved, and periodically monitored by the Ethics Committee of Mackay Memorial Hospital, Taipei, Taiwan (20MMHIS410e and 23MMHIS058e), which waived the requirement for informed consent. Questionnaire data, pressure–flow studies, and pressure–volume measurements from 23 female patients with SUI (<75 years) who completed a home-based PFMT + IVS program and underwent both pre- and post-treatment cystometry at a tertiary teaching hospital were retrospectively reviewed. Patients with non-SUI storage symptoms or incomplete pressure–volume loops were excluded. A total of 20 patients were included in the final analysis.

### 2.2 Interventions

Before initiating the therapy program, patients received instruction on pelvic floor anatomy and the technique for pelvic floor muscle contraction from a physical therapist in the hospital. Thereafter, patients independently performed the home-based exercise program. Biofeedback-assisted pelvic floor muscle training (PFMT) was conducted using a wireless module (MMS, Enschede, the Netherlands). Patients received auditory instructions and feedback via headphones and performed training twice weekly for 20 min per session. Intravaginal electrical stimulation (IVS) was delivered using the FemiScan system (Mega Electronics, Kuopio, Finland) with the following parameters: frequency 35 Hz, pulse width 250 µs, duty cycle 5 s on/10 s off, and current intensity set just below the patient’s maximal tolerable level (up to 100 mA). Each session lasted 20 min and was performed twice weekly. Patients underwent combined PFMT + IVS for 3 months.

### 2.3 Questionnaires

Symptom severity and quality of life were assessed using the Urogenital Distress Inventory-6 (UDI-6; score range 0–100, higher scores indicating greater symptom burden) and the Incontinence Impact Questionnaire-7 (IIQ-7; score range 0–100, higher scores indicating worse quality of life) (Skorupska et al., 2021).

### 2.4 Pressure–flow and pressure–volume studies

Pre- and post-treatment urodynamic assessments were performed 1–6 weeks before treatment initiation (mean = 24.95 ± 7.08 days) and 1–8 months after treatment completion (mean =180.10 ± 42.89 days), respectively. Studies were performed in accordance with International Continence Society guidelines (Schäfer et al., 2002; D'Ancona et al., 2019). Warm saline (37 °C) was infused into the bladder at a rate of 80 mL/min. The following parameters were recorded: vesical pressure (Pves), abdominal pressure (Pabd), detrusor pressure (Pdet), urinary flow rate (Flow), voided volume (Vvoid), infused volume (Vinf), and intravesical volume (Vive). Derived parameters included mean voiding pressure (Pvoid; mean Pdet during urine emission), voiding time (Tvoid; duration of urine flow), mean voiding flow rate (Fvoid; Vvoid/Tvoid), and mean voiding resistance (Rvoid; Pvoid/Fvoid). Pressure–volume analysis (PVA) was performed by plotting Pdet against Vive (Lau et al., 2023; Lau et al., 2024c; Peng et al., 2020). The trajectory-enclosed area (Apv), representing a surrogate measure of voiding energy expenditure, was quantified using ImageJ (LOCI, Madison, WI, USA).

### 2.5 Statistical analysis

Baseline characteristics, including age and the interval between treatment initiation and cystometric evaluation (pre- and post-treatment), were summarized using descriptive statistics. Data are presented as mean ± standard deviation (SD). Pre- and post-treatment comparisons were performed using the Wilcoxon signed-rank test. A p-value <0.05 was considered statistically significant.

## 3 Results

### 3.1 Patient database

The demographics the 20 female patients were: mean age, 55.10 ± 3.18 years; mean gravidity, 3.1; mean parity, 2.8. Comorbid medical conditions of these patients were hypertension (5 patients), diabetes (4 patients), hyperlipidemia (2 patients), and autoimmune hepatitis (1 patient). No patients had neurogenic bladder or used medications that might affect bladder function. Ten patients had a history of prior pelvic surgery including total hysterectomy (6 patients), Cesarean section (5 patients), dilation and curettage (1 patient). Pre- and post-operative urodynamic assessments were performed at 24.95 ± 7.08 days before the beginning and 180 ± 42.89 days after the completion of the intervention.

### 3.2 Improved UDI-6 and IIQ-7 scores

**FIGURE 1:**
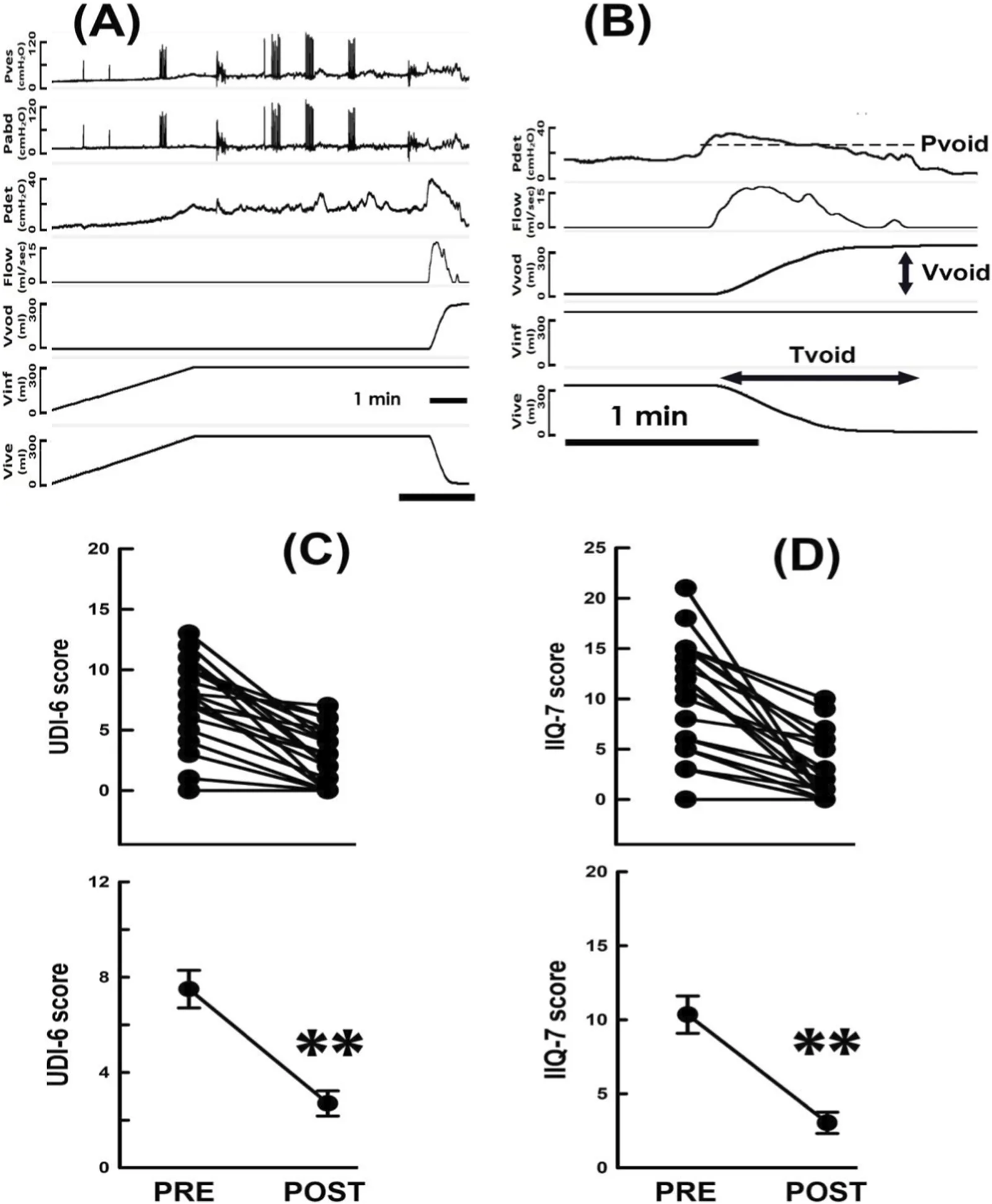
Urodynamic parameters and questionnaire outcomes. **(A)** Representative cystometric tracings showing vesical pressure (Pves), abdominal pressure (Pabd), detrusor pressure (Pdet), urinary flow (Flow), voided volume (Vvoid), infused volume (Vinf), and intravesical volume (Vive). **(B)** Quantified parameters derived from the lower five tracings in **(A)** indicated by the bar: voiding pressure (Pvoid), voided volume (Vvoid), and voiding time (Tvoid). **(C,D)** Individual (upper panels) and mean (lower panels) UDI-6 (**p < 0.001 vs. PRE; N = 20) and IIQ-7 (**p < 0.001 vs. PRE; N = 20) scores measured before (PRE) and after treatment (POST).

Prior to evaluating the objective effects of PFMT + IVS on voiding function, we first confirmed its clinical benefit on urinary continence by assessing symptoms and quality of life (QoL) using the UDI-6 and IIQ-7 questionnaires. Compared with pre-treatment values (PRE; Figure 1C), UDI-6 scores decreased in the majority of patients (19/20; 95%), resulting in a significant reduction in the mean UDI-6 score following PFMT + IVS (POST; p < 0.001; N = 20). Similarly, IIQ-7 scores decreased in 19 of 20 patients (95%; Figure 1D), with a significantly lower mean score observed post-treatment compared with pre-treatment (p < 0.001; N = 20).

### 3.3 Increased voiding work

**FIGURE 2:**
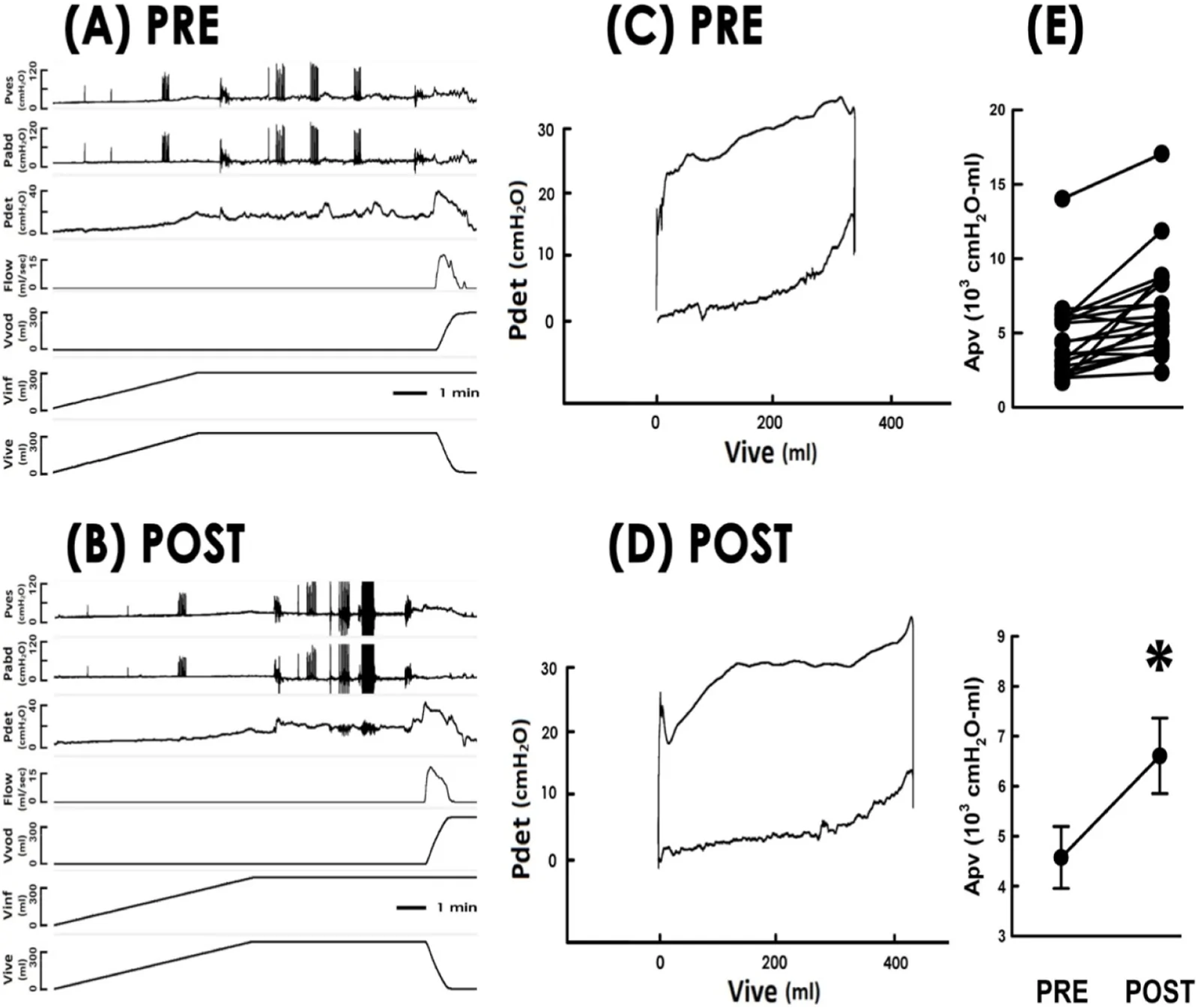
Urodynamic and pressure–volume analyses in response to treatment. **(A,B)** Representative cystometry tracings and **(C,D)** corresponding pressure–volume analyses from a patient with SUI obtained before and after PFMT + IVS (PRE and POST, respectively). **(E)** Individual (upper panel) and mean (lower panel) values of the pressure–volume loop–enclosed area (Apv; *p = 0.044 vs. PRE; N = 20). Vesical pressure (Pves), abdominal pressure (Pabd), detrusor pressure (Pdet), urinary flow (Flow), voided volume (Vvoid), infused volume (Vinf), and intravesical volume (Vive).

Having observed PFMT + IVS improved continence-related symptoms and QoL, we next examined its effects on voiding workload. Pressure–volume analyses (PVAs) were constructed from pre- and post-treatment cystometric data (Figures 2A,B, respectively) by plotting detrusor pressure (Pdet) against intravesical volume (Vive) (Figures 2C,D). Trajectories in each PVA formed a closed loop representing a complete voiding cycle. PFMT + IVS exerted minimal effects on the top, left, and bottom boundaries of the loop but shifted the right boundary rightward relative to pre-treatment, thereby increasing the loop-enclosed area (Apv), which reflects bladder voiding work. Consistent with this, Apv increased in most patients (18/20; 90%; Figure 4E), and the mean Apv was significantly higher post-treatment than pre-treatment (p = 0.044; N = 20).

### 3.4 Unaffected voiding pressure but increased voiding volume

**FIGURE 3:**
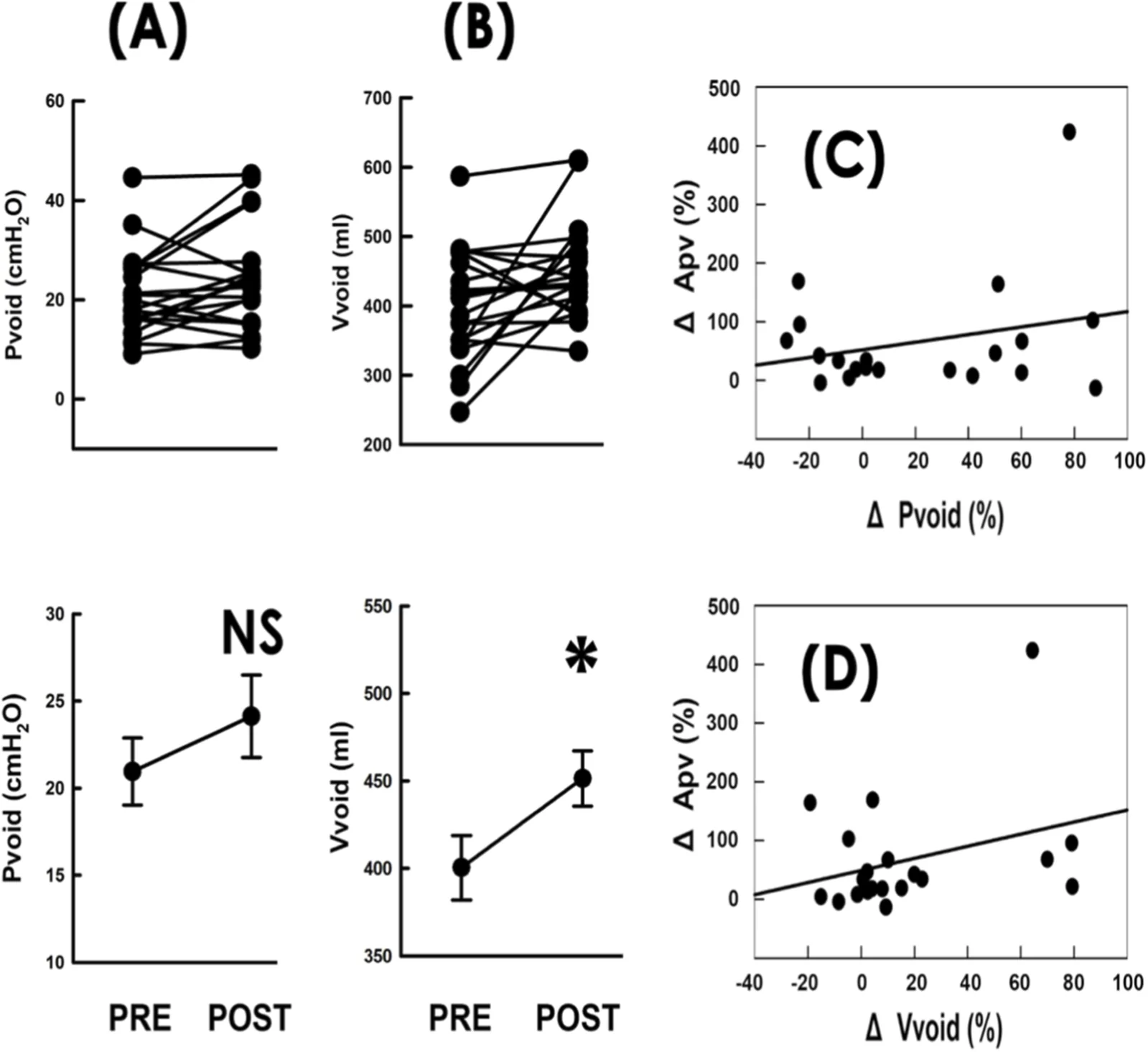
Apv-associated parameters in response to treatment. **(A,B)** Individual (upper panels) and mean (lower panels) values of voiding pressure (Pvoid) and voided volume (Vvoid), respectively, measured before (PRE) and after (POST) treatment. Pvoid was unchanged (NS, p = 0.306; N = 20), whereas Vvoid was significantly increased (*p = 0.042 vs. PRE; N = 20). **(C,D)** Bivariate correlations between treatment-induced changes in Apv (ΔApv) and changes in voiding pressure (ΔPvoid; r = 0.262, p = 0.264) or voided volume (ΔVvoid; r = 0.450, p = 0.046).

As Apv represents a pressure–volume integral, we next examined whether the observed increase in voiding work was attributable to changes in voiding pressure (Pvoid) or voiding volume (Vvoid). Cystometric data showed that PFMT + IVS did not consistently alter individual Pvoid values and did not significantly change the mean Pvoid compared with pre-treatment (Figure 3A; p = 0.306; N = 20). In contrast, Vvoid increased in most patients (15/20; 75%; Figure 3B), with a significantly higher mean value observed post-treatment than pre-treatment (p = 0.042; N = 20). These findings indicate that the increase in Apv was primarily driven by an increase in voiding volume rather than voiding pressure.

### 3.5 Correlated increments in voiding work and volume

We next evaluated the relationships between changes in voiding work and changes in pressure or volume by correlating treatment-induced changes in Apv (ΔApv) with those in Pvoid (ΔPvoid) and Vvoid (ΔVvoid). No significant correlation was observed between ΔApv and ΔPvoid (Figure 3C; r = 0.262, p = 0.264; N = 20). In contrast, ΔApv was significantly correlated with ΔVvoid (Figure 3D; r = 0.450, p = 0.046; N = 20), indicating that the increase in voiding work was associated with increased voiding volume rather than pressure.

### 3.6 Unaffected voiding resistance

**FIGURE 4:**
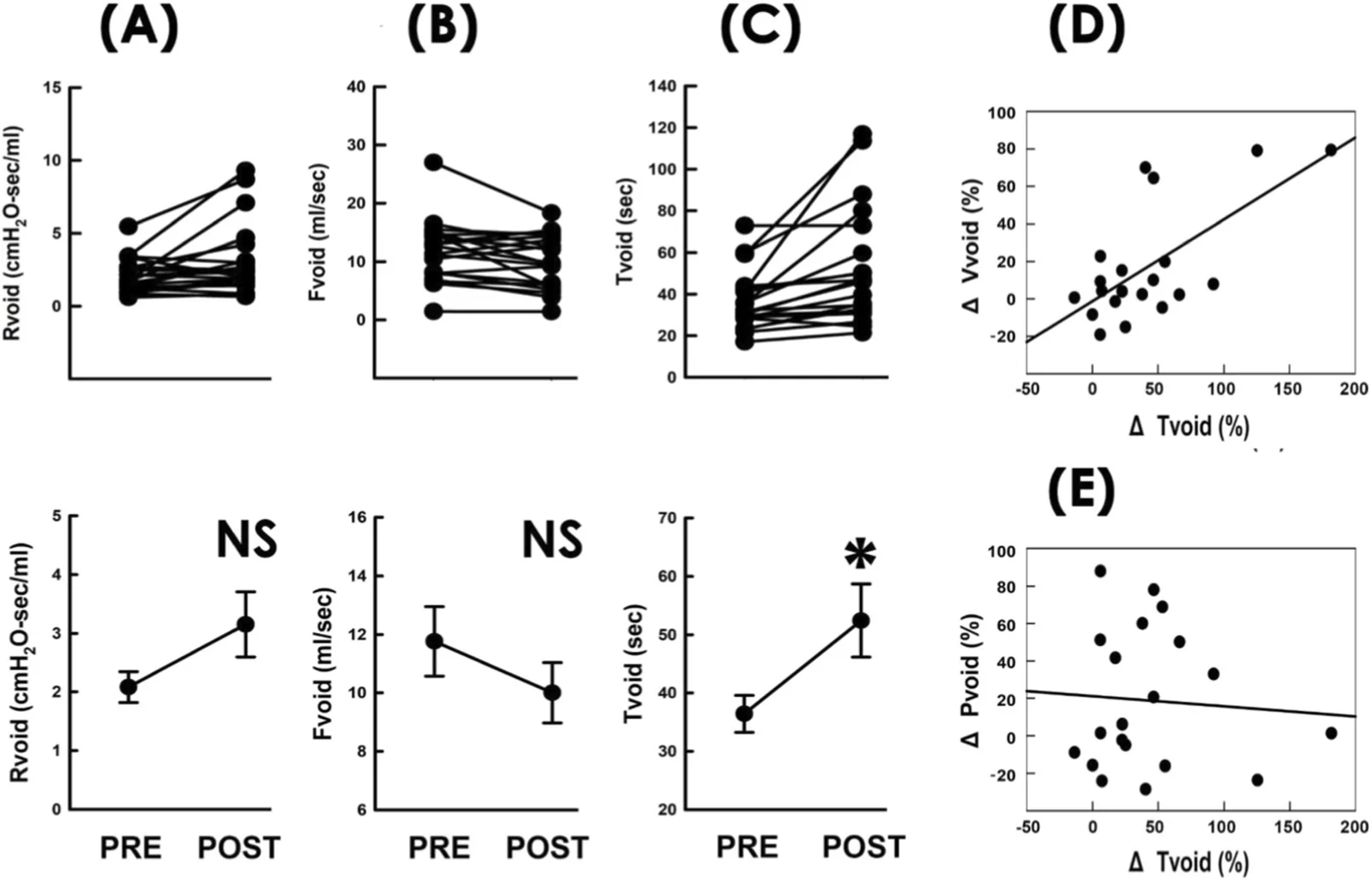
Resistance-associated parameters in response to treatment. **(A–C)** Individual (upper panels) and mean (lower panels) values of voiding resistance (Rvoid), voiding flow (Fvoid), and voiding time (Tvoid), respectively, measured before (PRE) and after (POST) treatment. NS p = 0.089 for Rvoid, NS p = 0.271 for Fvoid, *p = 0.028 for Tvoid vs. PRE; all N = 20. **(D,E)** Bivariate correlations between treatment-associated changes in voided volume (ΔVvoid; r = 0.665, p = 0.001) or voiding pressure (ΔPvoid; r = 0.068, p = 0.773) and changes in voiding time (ΔTvoid).

To further elucidate the mechanism underlying the increased voiding workload, we examined whether PFMT + IVS altered voiding resistance (Rvoid), as treatments for SUI are often intended to restore urethral resistance. Unexpectedly, urodynamic analyses showed that PFMT + IVS neither consistently affected individual Rvoid values nor significantly changed the mean Rvoid compared with pre-treatment (Figure 4A; p = 0.089; N = 20).

### 3.7 Unaffected voiding flow

As Rvoid is defined as Pvoid/Fvoid and neither Pvoid nor Rvoid was altered by PFMT + IVS, we next assessed its effect on voiding flow rate (Fvoid). Compared with pre-treatment values, PFMT + IVS did not consistently affect individual Fvoid values and did not significantly change the mean Fvoid (Figure 4B; p = 0.271; N = 20), indicating a negligible effect on voiding flow.

### 3.8 Prolonged voiding time

Given that PFMT + IVS increased Vvoid without affecting Fvoid (defined as Vvoid/Tvoid), we next examined voiding time (Tvoid). PFMT + IVS increased Tvoid in most patients (18/20; 90%; Figure 4C) and significantly increased the mean Tvoid compared with pre-treatment (p = 0.028; N = 20). These findings indicate that PFMT + IVS produced proportional increases in voiding volume and time, thereby maintaining an unchanged flow rate during voiding.

### 3.9 Correlated increments in voiding volume and time

Finally, correlation analyses demonstrated a strong association between treatment-induced changes in voiding volume and voiding time (ΔVvoid and ΔTvoid, respectively; Figure 4D; r = 0.665, p = 0.001; N = 20). In contrast, no correlation was observed between ΔPvoid and ΔTvoid (Figure 4E; r = −0.068, p = 0.773; N = 20). These findings indicate that the PFMT + IVS-associated increase in voiding volume, but not voiding pressure, was accompanied by a proportional increase in voiding time.

## 4 Discussion

Consistent with prior studies demonstrating the benefits of IVS (Stania et al., 2022), PFMT (Todhunter-Brown et al., 2022), and PFMT + IVS (Lau et al., 2024a) in patients with SUI, the present study showed that PFMT + IVS improved urinary continence. This improvement was evidenced subjectively by reduced symptom burden and enhanced quality of life on the UDI-6 and IIQ-7 questionnaires, and objectively by urodynamic and pressure–volume analyses demonstrating increased bladder storage capacity following treatment.

Beyond improvements in continence, pressure–volume analysis (PVA) revealed that PFMT + IVS selectively shifted the right border of the pressure–volume loop to the right, without altering the bottom, left, or top borders. This shift resulted in an increased Apv. Because Apv is a thermodynamic parameter that quantifies the work expenditure during voiding (Lau et al., 2023; Lau et al., 2024c; Peng et al., 2020), these findings suggest that the bladder adapts to the treatment-associated increase in voided volume by expending greater voiding work. Specifically, the rightward displacement of the loop’s right border—representing voided (or infused) volume per voiding cycle—indicates an increase in Vvoid, a finding corroborated by urodynamic measurements. In contrast, the relatively unchanged top border of the loop, which represents voiding pressure (Pvoid), suggests that PFMT + IVS exerted minimal influence on voiding pressure, a conclusion further supported by urodynamic data. Given that Apv is defined as a pressure–volume integral, these results indicate that the increased voiding work following treatment was primarily attributable to increases in Vvoid rather than changes in Pvoid. This speculation is reinforced by correlation analyses showing that treatment-associated changes in Apv correlated with ΔVvoid but not with ΔPvoid. Collectively, these findings indicate that PFMT + IVS-induced increases in voiding work are largely driven by enhanced storage capacity leading to greater voided volume.

Notably, although questionnaire outcomes, urodynamic parameters, and PVA consistently demonstrated increased storage volume after PFMT + IVS, outlet resistance during voiding remained largely unchanged that is supported by urodynamic analysis showing no significant alteration in Rvoid following treatment. Although this finding may appear counterintuitive, PFMT + IVS is known to strengthen pelvic musculature, thereby restoring outlet resistance during the storage phase to maintain continence (Peyronnet et al., 2025). The improved questionnaire scores and increased Vvoid observed in this study confirm that pelvic muscle strengthening provided sufficient outlet resistance during storage.

Conversely, because pelvic floor muscles is able to relax during voiding to reduce outlet resistance and permit urine flow (Clothier and Wright, 2018), we propose that the treatment-strengthened musculature retained—or even enhanced—its ability to relax appropriately during voiding. Consequently, patients exhibited improved continence during storage while maintaining unaltered voiding resistance. This proposal is supported by the observation that both Fvoid and Pvoid remained unchanged following intervention. If outlet resistance during voiding were increased, the bladder would be expected to compensate either by elevating Pvoid to preserve Fvoid or by maintaining Pvoid with a concomitant reduction in Fvoid. The simultaneous preservation of both Pvoid and Fvoid therefore indicates that Rvoid remained unchanged after PFMT + IVS. Given that Rvoid is calculated as Pvoid/Fvoid, these findings further confirm that PFMT + IVS did not alter voiding resistance. Together, these results suggest that PFMT + IVS not only improves continence during storage but also preserves effective pelvic muscle relaxation during voiding, ensuring adequate urine flow.

In parallel with the increase in Vvoid, urodynamic data in the current study revealed a corresponding increase in voiding time (Tvoid) following PFMT + IVS. Because Fvoid is calculated as Vvoid/Tvoid, the unchanged Fvoid observed in this study can be explained by proportional increases in both Vvoid and Tvoid. This interpretation is supported by bivariate analyses demonstrating a positive correlation between treatment-associated increases in Vvoid and Tvoid. Although the precise mechanisms underlying these synchronous increases remain unclear, the data suggest that, in response to enhanced storage capacity, the bladder requires a longer duration to empty a larger voided volume while maintaining a stable flow rate.

In contrast to PFMT + IVS, previous studies have shown that in SUI patients undergoing trans-obturator tape (TOT) repair, the implanted sling provides sufficient outlet resistance to restore continence during storage but simultaneously acts as a fixed physical barrier that impedes voiding (Lau et al., 2022). To overcome this increased resistance, the bladder adapts postoperatively by generating elevated voiding pressure. The mechanisms underlying the distinct bladder adaptations observed with PFMT + IVS versus TOT remain unclear. Potential explanations include greater resistance imposed by implanted slings compared with noninvasive muscle strengthening, or differences between a static exogenous barrier and dynamically controlled pelvic musculature capable of contracting during storage and relaxing during voiding. Importantly, TOT-associated increases in outlet resistance lead to elevated voiding pressure and increased voiding workload (Lau et al., 2022), indicating that more work is required to void a given volume. In contrast, the increased voiding workload associated with PFMT + IVS arises primarily from greater voided volume without a concomitant rise in voiding pressure; thus, the workload required to void a given volume remains relatively unchanged, provided that voiding time is extended. Consequently, for an equivalent daily voided volume, TOT repair may impose a greater long-term workload on the bladder than PFMT + IVS. Given evidence that chronic increases in bladder workload may contribute to detrusor dysfunction (Chow et al., 2021), PFMT + IVS may represent a therapeutic option with a lower risk of detrusor decompensation compared with TOT implantation.

It is worth noting that, with the advancement of minimally invasive therapies for SUI, Bulkamid, a homogeneous polyacrylamide hydrogel bulking agent, has emerged as an alternative treatment. Bulkamid is injected into the urethral submucosa to enhance urethral coaptation and thereby reduce involuntary urine leakage (Särkilahti et al., 2025). Because its mechanism of action differs from that of conservative pelvic floor muscle training, which improve urethral closure by strengthening the pelvic floor muscles, and from mid-urethral slings, which provide a supportive hammock beneath the urethra, future urodynamic studies evaluating bladder and urethral function following Bulkamid treatment may provide valuable mechanistic insights and help clinicians optimize treatment selection for patients with SUI.

This retrospective analysis of existing data has inherent limitations affecting both internal and external validity. The relatively small sample size may introduce measurement bias and limit generalizability. Because urethral pressure was not measured, this study lacks direct evidence that PFMT + IVS increased urethral pressure, which subsequently led to elevated bladder pressure during voiding. Therefore, future urodynamic studies incorporating urethral pressure measurements are warranted to test this proposed mechanism. Moreover, considering PFMT + IVS may improve continence by enhancing hypogastric nerve tone (Lau et al., 2024a), though direct assessment of neural activity in clinical practices remains challenging, the potential contribution of neural mechanisms to the therapeutic effects of PFMT + IVS warrants further investigation.

## 5 Conclusion

Unlike mid-urethral sling implantation, which increases outlet resistance and thereby elevates voiding pressure and overall voiding workload, conservative PFMT + IVS provides sufficient resistance during the storage phase while allowing effective relaxation during voiding. Consequently, it exerts minimal effects on voiding resistance, voiding pressure, and urinary flow. Although total thermodynamic work per voiding cycle increased due to larger post-treatment voided volumes, the work required per unit volume of urine remained unchanged.

## Data Availability

The raw data supporting the conclusions of this article will be made available by the authors, without undue reservation.
